# Function of phosphorylation of NF-kB p65 ser536 in prostate cancer oncogenesis

**DOI:** 10.18632/oncotarget.3366

**Published:** 2015-01-31

**Authors:** Li Zhang, Longjiang Shao, Chad J. Creighton, Yiqun Zhang, Li Xin, Michael Ittmann, Jianghua Wang

**Affiliations:** ^1^ Department of Biochemistry and Molecular Biology, College of Basic Medical Sciences, Third Military Medical University, Chongqing, China; ^2^ Department of Pathology and Immunology, Baylor College of Medicine and Michael E. DeBakey Deptartment of Veterans Affairs Medical Center, Houston, Texas, USA; ^3^ Duncan Cancer Center Division of Biostatistics, Baylor College of Medicine, Houston, Texas, USA; ^4^ Department of Molecular & Cellular Biology, Baylor College of Medicine, Houston, Texas, USA; ^5^ Department of Medicine, Baylor College of Medicine, Houston, Texas, USA

**Keywords:** Prostate cancer, TMPRSS2/ERG, NF-κB, AKT, CCL2

## Abstract

Majority of prostate cancer (PCa) patients carry TMPRSS2/ERG (T/E) fusion genes and there has been tremendous interest in understanding how the T/E fusion may promote progression of PCa. We showed that T/E fusion can activate NF-kB pathway by increasing phosphorylation of NF-kB p65 Ser536 (p536), but the function of p536 has never been studied in PCa. We report here that active p536 can significantly increase cell motility and transform PNT1a cells (an immortalized normal cell line), suggesting p536 plays a critical role in promoting PCa tumorigenesis. We have discovered a set of p536 regulated genes, among which we validated the regulation of CCL2 by p536. Based on all evidence, we favor that T/E fusion, NF-kB p536 and CCL2 form a signaling chain. Finally, PNT1a cells (not tumorigenic) can form tumors in SCID mice when overexpressing of either wild type or active p65 in the presence of activated AKT, demonstrating synergistic activities of NF-kB and AKT signals in promoting PCa tumorigenesis. These findings indicate that combination therapies targeting T/E fusion, NF-kB, CCL2 and/or AKT pathways may have efficacy in T/E fusion gene expressing PCa. If successful, such targeted therapy will benefit more than half of PCa patients who carry T/E fusions.

## INTRODUCTION

In spite of recent progress, prostate cancer (PCa) is still the second leading cause of cancer death in US men. PCa can be cured only if detected at an early localized stage using radical prostatectomy (RP) or radiation therapy. For recurrent or metastatic PCa, standard androgen ablation therapy usually results in initial regression of the disease. However, almost all patients will fail this therapy in the long run and develop “androgen-independent” (AI) or “castrate-resistant” PCa (CRPC), the lethal phenotype of PCa. From the clinical perspective, PCa patients even with identical stage and grade of primary localized tumor may develop very variable clinical outcomes due to the underlying differences at the molecular level. Characterization of differences in molecular signature among different patients is critical for designing personalized medicine.

A milestone discovery of PCa research in recent years was the identification of recurrent fusion of the androgen-regulated TMPRSS2 gene to the ERG gene in the majority of PCa lesions. The TMPRSS2/ERG (T/E) fusion gene is present in 40-60% of PCas, and is thus the most common genetic lesion in PCa [1-8]. As shown by Dr. Balk's group, its expression is even restored in CRPC [4]. The high frequency of this alteration and its important role in PCa tumor biology makes it an outstanding therapeutic target in PCa. We have shown that stable shRNA expression or siRNAs that specifically target T/E fusion transcripts significantly decrease tumor growth *in vivo* [9, 10]. Efforts are underway in our lab to search for key pathways involved in tumorigenesis of T/E fusion expressing PCa. We have shown that the T/E fusion gene can activate the NF-kB pathway by increasing phosphorylation of NF-kB p65 Ser536 (p536) [11].

Aberrant regulation of NF-kB pathway is believed to be a major event contributing to malignant transformation and progression of PCa [12-16]. Studies have shown that NF-kB plays an important role in PCa growth, survival, angiogenesis, tumorigenesis and metastatic progression [13]. Immunohistochemistry (IHC) studies have shown that increased nuclear NF-kB staining is a strong independent predictor of biochemical recurrence following RP [14, 17]. Studies also show the crosstalk of NF-kB and AR signaling [18]. Due to its highly activated status in PCa and its role in promoting PCa to CRPC and metastatic cancer [19], NF-kB has been proposed as a potential target for therapeutic intervention for CRPCs [12, 18]. NF-kB transcription factors have five members in mammals, among which p65 and p50 are the most abundant in the cell. Without stimulation, NF-κB is inactivated in the cytoplasm by IκB inhibitory proteins. Upon activation, IkBs are phosphorylated by IkB kinases and degraded via ubiquitination and proteasomal degradation [20], which allows the translocation of p65 to the nucleus where it regulates the transcription of a wide variety of genes involved in cell survival, invasion and metastasis. Many key genes regulated by this transcription factor including BCL-2, cyclin D1, MMP-9, IL-6, IL-8 and VEGF [15]. P65 consists of a DNA-binding and dimerization domain (RHD), nuclear localization signal (NLS) and transactivation domains (TA1 and TA2). The c-terminal 30 amino acids (TA1 domain) comprise the most important transactivation domain and NF-kB transactivation is regulated by multiple phosphorylations in this domain. The p536 phosphorylation site is located in TA1 domain and is conserved in human, mouse, chicken. Phosphorylation of p65 has been shown by many groups to enhance p65 transcriptional activity (see reviews [21] and [22]), but its function and regulation has never been studied in PCa.

We have shown that p536 is present in majority of PCa, is correlated with ERG expression and that ERG can significantly enhance phosphorylation of p65 at Ser536 *in vitro*. Since NF-kB pathway, especially p536 signaling is highly activated in T/E fusion expressing PCa, we hypothesize that p536 plays a critical, yet only partially understood, role in progression of PCas expressing the T/E fusion gene and that targeting NF-kB signaling in addition to traditional therapies may be more efficacious in this subgroup of PCa. Supporting this idea, we have shown that NF-kB inhibitor can significantly inhibit T/E fusion expressing PCa cell growth *in vivo* [23].

We have successfully generated a p65 mutant where serine 536 was substituted with alanine (S536A) such that it cannot be phosphorylated at Ser536 and cannot carry out Ser536 phosphorylation dependent functions [24], designated as nonphosphorylatable (NP) p65 mutant. We also generated two active mutants, S536D and S536E, which are the phosphomimetic mutant that mimics phospho-p65 in its activity and can be detected by anti-phospho-p65 antibody. In particular, S536D mutant has been shown to enhance p65 transcriptional activity and regulate specific genes downstream of the NF-kB pathway [25, 26]. Using these mutants, we now show that active p536 can significantly increase cell invasion through matrigel and transform PNT1a cells (an immortalized prostate normal cell line), suggesting p536 play a critical role in promoting PCa tumorigenesis. Our microarray study discovered a set of p536 regulated genes. Among them, we validated the regulation of CCL2 by p536, indicating that upregulated CCL2 signaling may be the cause of increased cell motility/invasion in T/E fusion as reported earlier [9] and/or in p536 overexpressing cells as shown in this study. Finally, PNT1a cells, which are not tumorigenic, can be induced to form tumors in SCID mice by overexpression of either wild type (WT) p65 or p65 S536E in the presence of myristoylated AKT demonstrating synergistic activities of NF-KB and AKT signals in promoting PCa tumorigenesis.

## RESULTS

### Biological effects of p536 expression *in vitro*

**Figure 1:**
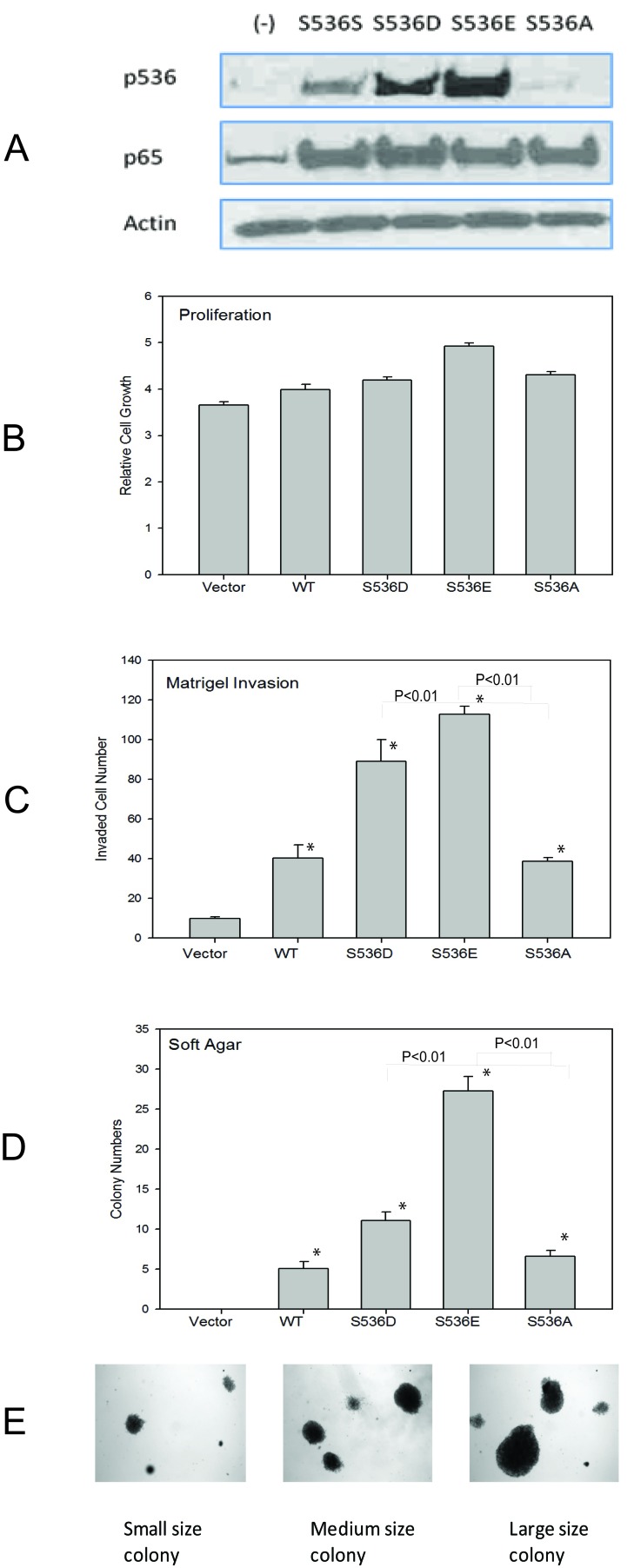
Biological effects of p536 in prostate cell line A. PNT1A cells overexpressing WT p65 (S536S), active p536 (S536D and S536E) and nonphosphorylatable S536A were infected with Lentiviruses carrying these expression constructs and negative control and stable expressors selected and pooled. Western blot shows similar level of total p65 expression and expected p536 expression in these cell lines; B. PNT1a cells as described in A were plated into 96-well plate and growth rates were compared using MTT assay. The absorbance at 570nm was recorded at 7 days after seeding using 96-well plate reader. Representative result of mean with standard deviation of triplicates is shown. C.PNT1a cells as described in A were plated into matrigel chambers. 48h later, cells on the opposite side of the membrane were stained and counted. Experiment was repeated twice. Representative result of mean with standard deviation of triplicates is shown. D. PNT1a cells generated in A were plated in soft agar. Colony formation was evaluated by counting. Mean with standard deviation of triplicates of three times is shown. E. Different sizes of colonies formed are shown. * was indicated a statistically significant result when compared to control group. P values were shown on the top of bars between active and NP p536 groups.

As we have shown previously [27], among all commonly used prostate cell lines, only VCaP cells show high level of p536 expression. To study the function of p65 ser536 phosphorylation, we tried to overexpress active or nonphosphorylatable p536 in VCaP cells, but due to the endogenous high level of p536 expression, we could not establish stable cell lines with significant p536 expression changes. Therefore, we choose another androgen dependant PCa cell line LNCaP and the immortalized but nontumorigenic prostatic epithelial cell line PNT1A. We established both PNT1a and LNCaP stable cell lines expressing WT p65 (S536S), p65 S536D (active p536), S536E (active p536), S536A (NP p635) or control. As shown in Fig. 1A, both p65 expression was similar in cell lines established with various expression constructs while p536 phosphomimetic expression (recognized by anti-p536 antibody) was high in the appropriate cell lines and no increase in p536 expression was seen in cells expressing the nonphosphorylatable S536A construct. A similar result was seen in LNCaP cell groups (data not shown). S536E group shows stronger signaling than S536D group by anti-p536 in both PNT1a and LNCaP group. Growth rates were tested and compared among all groups. After 7 days, there were only about 20-35% growth increase in WT and S536 D/E/A overexpression groups versus control groups in PNT1a cell lines. One representative result is shown in Fig. 1B. A similar result was seen in LNCaP cell groups (data not shown). Therefore, both WT p65 and active S536D/E alone did not show significant effects on cell growth *in vitro*.

Increased cell motility is one of the characteristics associated with malignancy and is involved in the genesis of metastatic disease. Therefore, as a measure of cell motility and invasiveness, we assessed the ability of cells expressing WT p65, S536D, S536E or NP p65 to invade through Matrigel (Fig. 1C). Cells expressing the WT p65, active or NP p65 had significantly higher invasiveness through Matrigel when compared with the control cells (P<0.05, T-test), with significant higher activities seen in active p65 groups compared to either WT p65 or NP S536 groups (P<0.01, T-test), indicating Ser536 phosphorylation promotes invasion. NP S536A showed similar activity as wild type p65, suggesting functions of p65 independent of Ser536 phosphorylation p536 are also involved in increased invasion activities.

We next tested colony formation, a hallmark of tumorigenesis, using PNT1a cell lines. PNT1A cells are immortalized but not fully transformed and do not form colonies in soft agar. As shown in Fig. 1D, overexpression of WT p65 and both active p536 as well as S536A lead to colony formation in soft agar. There were many more colonies in the S536D and S536 E groups than WT p65 group (significant different, P<0.01, T-Test). The colony sizes are also much bigger in the S536D and S536E groups compared to WT p65 and S536A groups. The size and number of colonies in S536A group were quite close to the ones in WT p65 group. Representative samples of different sizes of colonies formed are shown in Fig. 1E. The increased soft agar colony formation in NP p65 cell lines is probably due to increased total p65 expression, implying that p65 activities other than those dependent on p536 also contribute to colony formation. As a positive control, we used PNT1a cells expressing Huntington interacting protein-1 [28] which did form colonies in soft agar (data not shown).

### Regulation of a set of specific NF-kB target genes by p65 Ser536 phosphorylation

**Figure 2:**
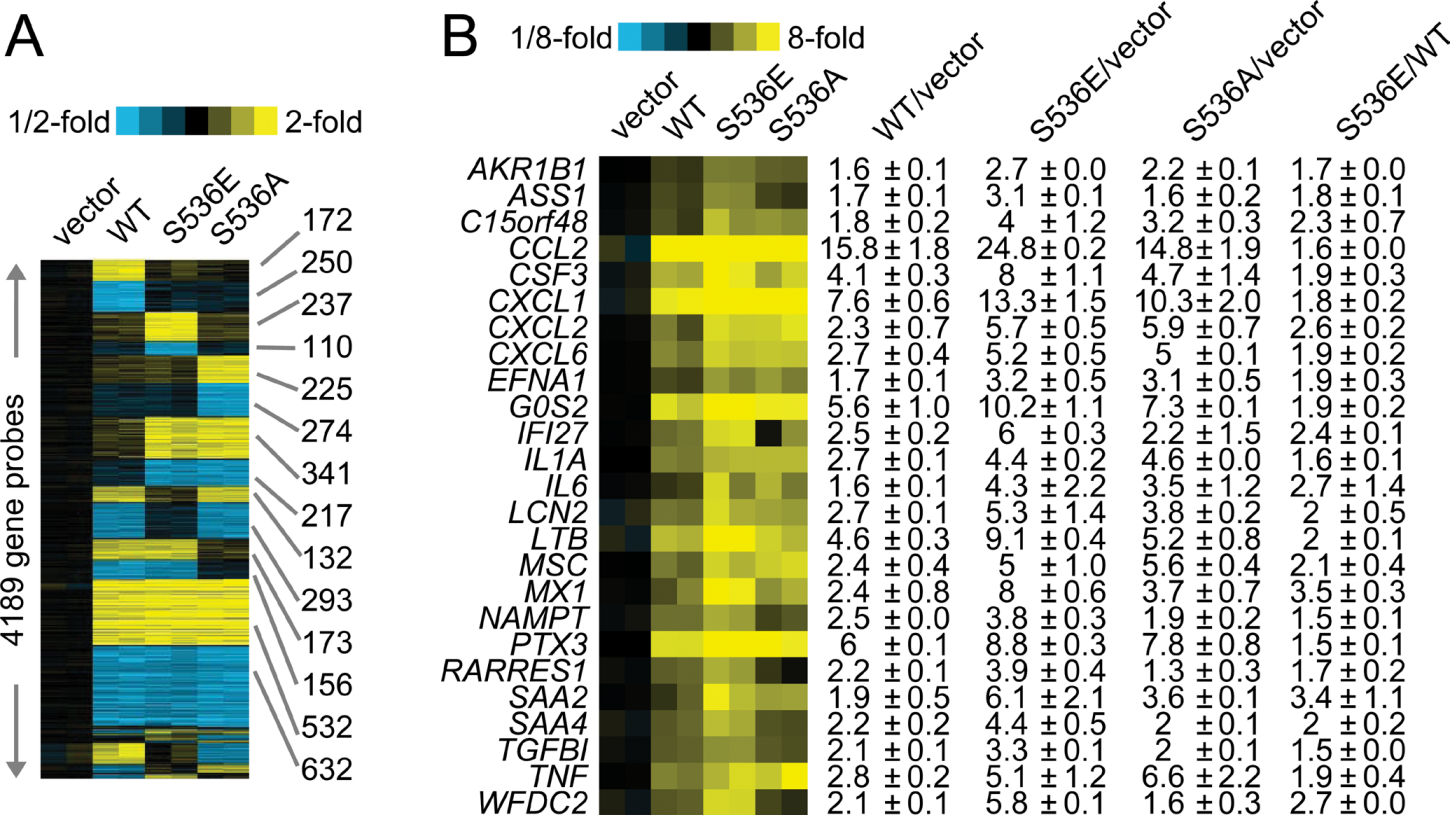
Microarray data of p536 specific regulated genes Gene expression microarray studies of biological duplicates were performed on control (“vector”), WT p65-(“WT”), active S536E-(“S536E”) and nonphosphorylatable S536A- (“S536A”) PNT1a cells, using Agilent G3 human 8x60k whole genome expression microarrays. A. Expression heat map (rows: genes; columns: profiled samples; yellow: high expression) for 4189 gene probes found differentially expressed (fold change>1.4 or <1/1.4 for each comparison profile vs each control profile) for any of the 3 p65 groups (WT, S536E, S536A) vs vector control. B. Heat map representation of a top set of 25 genes differentially expressed in S536E p65 group compared to WT p65 group (1.4-fold each WT p65 profile vs each pCDH profile, 1.4-fold each S536E profile vs each pCDH profile, and 1.4-fold each S536E profile vs each p65 profile); corresponding fold changes for these genes in each cell group are provided off to the right.

**Figure 3:**
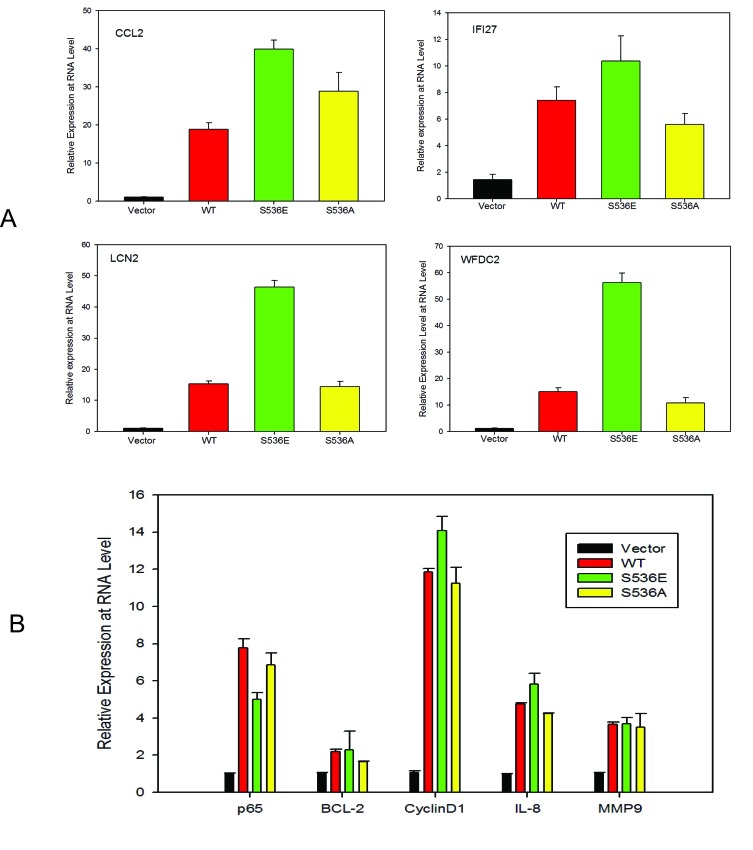

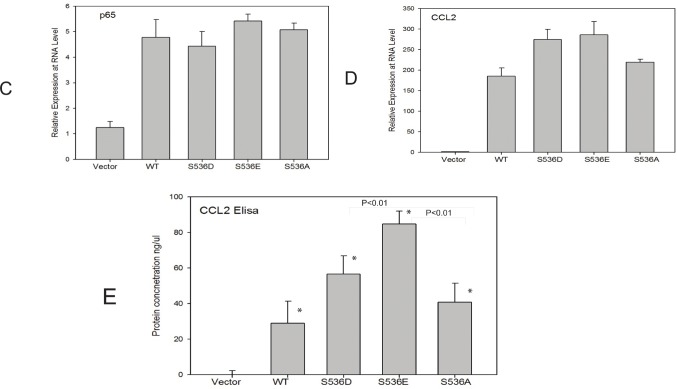
p536 regulated gene expression validation A. Quantitative RT-PCR validation of selected genes in vector-, WT p65-, S536E- and S536A-PNT1a cells from the list of heat map. Relative expression of CCL2, IFI27, LCN2 and WFDC2 are shown. Mean with standard deviation of triplicate determinations is shown. β-Actin was used for normalization; B. Quantitative RT-PCR validation of well known p65 regulated genes of BCL-2, cyclinD1, IL-8 and MMP9 in PNT1a cell lines; Mean with standard deviation of triplicate determinations is shown. β-Actin was used for normalization; C-D. Total p65 and CCL2 expression are accessed by real-time PCR in LNCaP cells, + vector, +WT p65, +S536D, +S536E and +S536A; β-Actin was used for normalization; E. Secreted CCL2 proteins in culture medium by LNCaP cell lines were detected by Elisa. Experiment was repeated three times. Mean with standard deviation of triplicate is shown. * was indicated a statistically significant result when compared to control group. P values were shown on the top of bars between active and NP p536 groups.

To further understand the underlying molecular mechanisms of increased invasiveness and soft agar colony formation in S536E p65 expressing cells, microarray studies of biological duplicates were performed on control, WT p65-, S536E- and S536A-PNT1a cells, using Agilent G3 human 8x60k whole genome expression microarrays to identify the effector genes that may be responsible for phenotypic differences observed among groups. With the loess normalized data, we found 4189 gene probes significant with fold change>1.4 (or <0.7) for any of these 3 comparisons of WT p65 vs vector, S536E vs vector, or S536A vs vector (Fig. 2A). Many of these genes have been consistently reported as NF-KB/p65 regulated genes [29, 30] involved in immune response, inflammation, growth and/or cell motility, such as IL8 and IL6 (cytokine/chemokine); ICAM (cell adhesion molecule); CSF and VEGF (growth factors; and c-myc and IRF (transcription factors). Microarray data also revealed a top set of 25 genes differentially regulated between cells expressing WT and S536E p65 (heat map in Fig. 2B), many of which are often implicated in inflammation and cancer. For example, genes IL-1α, IL6, and CCL2 belonging to the cytokine/chemokine family, TGFβ has been previously reported to be regulated by NF-KB/p65 [29], and CSF3 is a reported growth factor. Selected genes including CCL2, WFDC2, LCN2 and IFI27 were confirmed in PNT1a cell RNAs by Q-RT-PCR (Fig. 3A). They were all significantly upregulated in S536E cells. We also confirmed several genes which are known p65 target genes but not specifically regulated by this Ser536 phosphorylation as shown in Fig. 3B. CCL2 (also called MCP-1) is a potent regulator of PCa cell migration and proliferation [31-34], and we have shown previously that NF-kB inhibitor can specially target its expression [23], therefore it was chosen for further investigations of its regulation by p536 in LNCaP cells. Total p65 expression levels are shown in Fig. 3C. As shown in Fig. 3D, CCL2 RNA expression was much higher in active S536D and S536E groups than in WT p65 and S536A group in LNCaP cells. Similarly, much more secreted CCL2 were detected in LNCaP cell culture medium from active S536D/S536E groups than WT p65 and S536A group (Fig. 3E, p<0.01, T-test). During our validation of microarray data by Q-RT-PCR, we found some genes' expression changes are cell context dependant. For example, IL8, a well known p65 targeted gene; its expression was highly upregulated in active p65 groups compared to WT p65 and S536A group in LNCaP cells; while its expression are highly upregulated in all p65 groups to similar levels in PNT1a-WT p65 cells. Therefore, there are definitely different responses to Ser536 phosphorylation in different cellular contexts.

### NF-kB ser536 phosphorylation plays a role in regulating CCL2 expression

**Figure 4:**
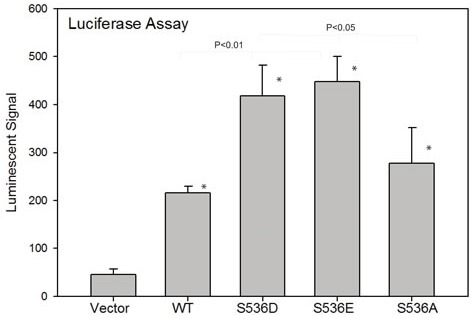
p536 regulates CCL2 promoter activities by luciferase reporter assay To determine the effect of ser536 phosphorylation on CCL2 expression, we co-transfected 293t cells with pGL3-CCL2 promoter vector and different p65 expression plasmids as well as control vectors. Luciferase activities in each group are shown. Mean with standard deviation of triplicate is shown. Experiments were repeated three times. * was indicated a statistically significant result when compared to control group. P values were shown on the top of bars between active p536 groups vs WT or active p536 groups vs NP p536 group.

Since evidence suggests CCL2 is a direct transcriptional target of NF-ΚB [35], we hypothesized that such Ser536 phosphorylation may have a direct impact on CCL2 expression. The CCL2 promoter contains 6 possible NF-kB binding sites. In order to determine the effect of Ser536 phosphorylation on CCL2 expression, we co-transfected 293t cells with pGL3-CCL2 promoter vector and different p65 expression plasmids as well as control vector. As shown in Fig. 4, luciferase activities were highly significantly upregulated in all p65 expression groups compared to control group, with the highest levels seen in S536D and S536E groups, confirming that p65 Ser536 phosphorylation has a potent impact on activating CCL2 promoter activity. Again, the activity level in S536A group was much higher than control group and similar as the level in WT p65 group, indicating that p65 activities not affected by Ser536 phosphorylation are also involved in regulating CCL2 expression.

**Figure 5:**
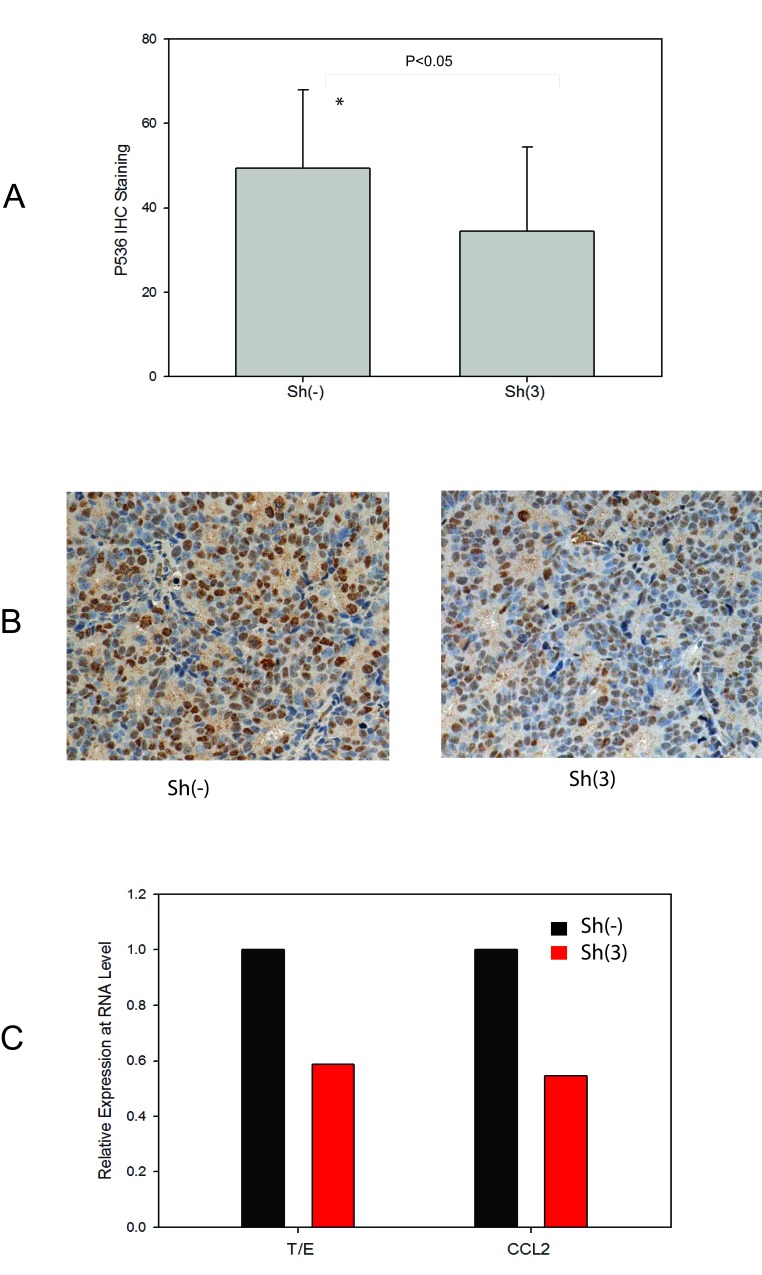
CCL2 as a p536 targeted gene confirmed in VCaP xenograft model with T/E fusion knock down A. Expression of p65 phospho-Ser536 in VCaP xenograft tumors without sh(−) / with sh(3) T/E fusion knockdown. IHC study using anti-p65 Ser536 antibody was performed using tumor slides and quantitated using a 0-3+ quantitation scale as described. Cases with the indicated staining indices for p65 phospho-Ser536 by IHC were scored and analyzed. Average expression levels are shown. The difference of p536 staining is significantly decreased in fusion knock down group compared to control group (P<0.05, T-Test); B. Representative p536 staining are shown. Left panel is one of sh(−) tumor with staining index scored as +3 (high expression); right panel is one of sh(3) tumor with staining index scored as +2 (moderate expression). C. Quantitative RT-PCR validation of T/E fusion and CCL2 using RNAs from both VCaP tumor groups.

To further confirm the correlation of Ser536 phosphorylation with CCL2 expression, we also accessed their expression levels in VCaP xenograft tumor samples we collected previously [9]. As shown in Fig. 5A, p536 expression was significantly decreased in T/E fusion knock down group (P<0.05, T-test). Representative p536 IHC staining of high/moderate expression in these VCap xenograft tumors collected are shown in Fig. 5B. CCL2 expression was also significantly lower in sh3 group than sh (−) group by real-time PCR (Fig. 5C). As we reported previously [23] Celastrol, a novel p536 inhibitor, can also significantly target CCL2 expression. Thus, we conclude that NF-kB p65 Ser536 plays a significant role in regulating CCL2 expression and the T/E fusion, NF-kB p536 and CCL2 may form a signal pathway that promotes tumor progression.

### Synergistic activities of both AKT and p536 in promoting tumor growth *in vivo*

**Figure 6:**
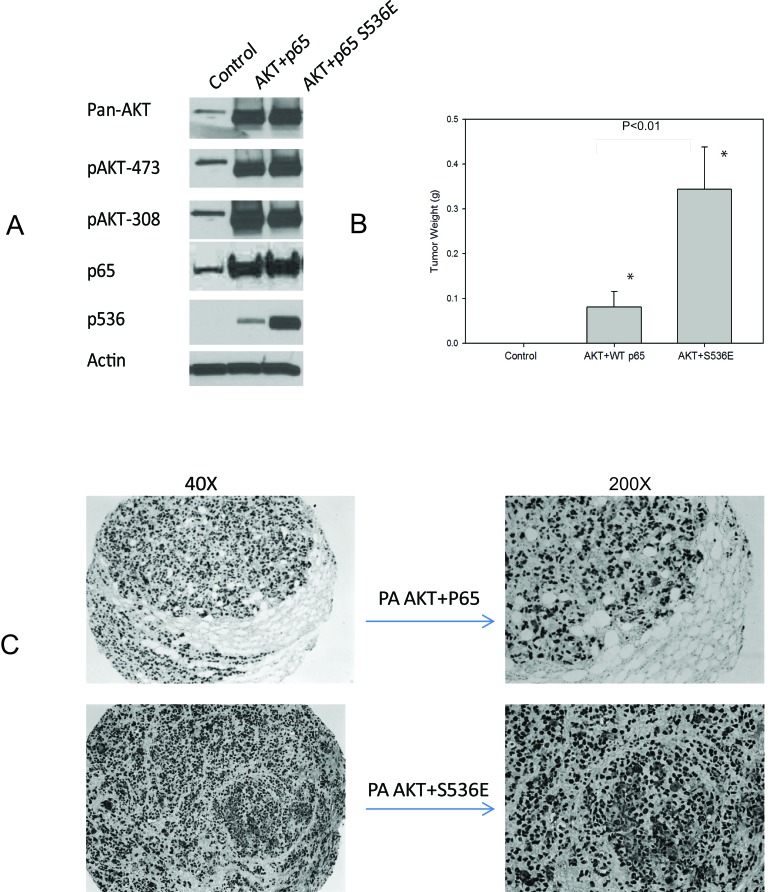
p65 Ser536 phosphorylation promotes tumor growth *in vivo* A. We introduced the myristoylated AKT into WT p65 and S536E PNT1a cells. Cells after sorting and before injections were lysed and expression levels of makers are shown by western blot; B. One million cells were injected into each frank of mice with matrigel. Mice were sacrificed at end of two months. Mean tumor weight with standard error of each group is shown in figure. The difference of tumor weight and tumor incidence between AKT+S536E group and other two groups were statistically significant (P<0.01, T-Test); C. IHC show positive SV40 staining indicating these tumors were indeed from PNT1a cells.

In initial experiments, we sought to determine if PNT1a cells overexpressing p536 mutant could grow in SCID mice since they did form colonies in soft agar. We had three experimental groups of PNT1a, PNT1a-WT p65 and PNT1a-p65 S536E. Repeated twice, they all failed to survive in SCID mice by subcutaneous injection with Matrigel. Therefore, we introduced the myristoylated AKT, which is a constitutively active Akt and well known to promote PCa growth, into these PNT1a cells, expecting to see certain synergistic activities. As shown in Fig. 6A, cells after sorting and before injection were lysed and expression levels of activity associated proteins assessed by western blot. Each group presents similar total AKT and phosphorylated AKT levels as well as expected p536 expression levels. As shown in Fig. 6B, no tumor was found in PNT1a cell group, small tumors were found in AKT+WT P65 group with low tumor incidence ( 6/20=30%); while AKT+S536E group showed much bigger tumors (average weight 0.344g) and much higher tumor incidence (16/20=80%). The difference of tumor weight and tumor incidence between AKT+S536E group and other two groups were statistically significant (P<0.01, T-Test), showing phosphorylation of p65 Ser536 play an important role in promoting tumor cells growth *in vivo*. As shown in Fig. 6C, we confirmed that these tumors collected were indeed SV40 positive PNT1a cells.

## DISCUSSION

Our group and others have carried out extensive studies of the incidence of T/E fusions in PCa, the various expressed different isoforms expressed as well as their biologic functions in PCa during the past a few years [2, 9, 10, 36, 37]. Due to the high frequency of such genetic rearrangements in PCa, patients carrying T/E fusions form the largest subgroup of PCas, and there is an urgent need to discover novel treatment options for this subgroup of patients. We have shown that knockdown of T/E fusion is a viable therapeutic approach [9, 10]. But they do not completely eliminate the tumor growth, suggesting some other signaling pathways and/or factors must also play a role there. Therefore, there has been considerable interest in understanding the mechanism by which the T/E fusion may promote progression of PCa.

We have shown previously that NF-kB signaling, via phosphorylation of NF-kB p65 Ser536, is highly activated in these T/E fusion expressing PCa cells and the absence of p536 is associated with decreased biochemical recurrence [36]. Aberrant regulation of NF-kB pathway is believed to be a major event contributing to malignant transformation and progression of PCa [14]. Therefore, we hypothesize that p536 may play a critical role in tumorigenesis in PCas bearing these T/E fusions, which has never been explored before. Our data show that active p65 Ser536 significantly promotes cell invasion through Matrigel with no obvious growth advantage seen. Most importantly, they can transform PNT1a immortalized normal prostate cells in a soft agar assay. The nonphosphorylatable form of p65 S536A group seemed to retain WT p65 basal activities, indicating that other activities of p65 not dependent on Ser536 phosphorylation also play a role in transformation. These biological activities are consistent with the oncogenic activity of the T/E fusion gene in prostatic epithelial cells and indicate the T/E gene fusion is driving neoplastic progression in part via phosphorylation of NF-kB p65 Ser536.

Due to the importance and complexity of the cellular processes regulated by NF-κB pathways, elucidating and characterizing NF-κB p536 regulated gene set will provide important insights into the role of this phosphorylation as well as identify potentially promising drug targets. Among all genes discovered by microarray study, CCL2 is the one that we are particularly interested in. CCL2 is a well known chemokine which plays a role in the clinical progression of several solid tumors including PCa [31-33, 38]. It regulates the recruitment of monocytes/macrophages and other inflammatory cells to the sites of inflammation through CCR2 receptor. Elevated CCL2/CCR2 expression was demonstrated in PCa malignancies when compared to benign prostatic tissues [32, 39]. CCR2 mRNA and protein are differentially expressed by PCa cells [31]. There is a growing body of research studies suggest that CCL2 can promote PCa cell proliferation, survival, migration and metastasis, as well as tumor angiogenesis [31-34, 38, 40-42]. Beside these direct impacts on cancer cells, the major influential role of CCL2 in the development and progression of tumor is its interaction with a variety of host cells in the microenvironment, including vascular endothelial cells, inflammatory cells and bone marrow cells (osteroblasts and endothelial cells). Importantly, CCL2 has been clearly shown to be a prominent modulator of metastatic growth in the bone microenvironment [31, 32, 43, 44], the most common site of PCa metastasis. The various functions of CCL2 promoting tumorigenesis make the CCL2 an attractive therapeutic target for PCa treatment. Studies have successfully shown that targeting CCL2 signaling using neutralizing antibody to CCL2 can significantly inhibit PCa tumor growth and migration *in vivo* including both VCaP (the only endogenously T/E fusion expressing PCa cell line) subcutaneous and intratibial injection models [39, 45]. As we reported earlier, CCL2 is upregulated in T/E fusion expressing PNT1a cells [11] which could result from the upregulated p536 activity in these cells. In this study, we confirmed that the p65 ser536 phosphorylation do play a role in regulating CCL2 expression in PCa cells. We also confirmed that when knock down T/E fusion in VCaP subcutaneous model as a therapy strategy, both p536 and CCL2 expression was significantly decreased [9]. In concordance with this, Celastrol, a novel p536 inhibitor, can also significantly inhibit VCaP tumor growth *in vivo* and target CCL2 expression [23]. Based on evidence presented above, we favor the concept that T/E fusion, NF-kB p536 and CCL2 may form a signaling chain. The elevated expression of p536 and CCL2 could result more aggressive behavior of cells as seen in these T/E fusion expressing cells. Since targeting T/E fusion alone cannot stop tumor growth completely, in combination of other NF-kB inhibitor and/or blocking CCL2 pathway may be an efficacious approach for this major group of PCas carrying T/E fusion.

Although we show here the strong biologic function of p536 in prostate cells, the mechanism of how p65 S536 is turned on in T/E fusion expressing PCa cells remain unknown. One possible mechanism is through TLR4 protein which can activate the NF-kB pathway and increase p536 when activated. As we reported earlier [11], we showed the decreased p536 when TLR4 was knocked down in PNT1a cells expressing the T/E fusion. But the detailed mechanism by which TLR4 activates p536 needs further investigation. In addition p65 Ser536 is the target of many kinases. There were multiple p65 Ser536 kinases have been described including IKKβ, RSK1, IKKα, IKKε and NAK [46-49]. But none of them was systematically studied in prostate system especially in T/E fusion expressing PCa. Whether these kinases involved in phosphorylation of p536 in T/E fusion expressing cells needs to be further analyzed.

It has been clear in the past a few years that T/E fusion play important function promoting PCa progression. However, expression of the T/E fusion alone cannot fully transform prostatic epithelial cells, implying the involvement of other signaling pathways. There is significant evidence from both correlative studies in human PCa and mouse models that PTEN loss / activated AKT signaling [3, 50-52] can cooperate with the T/E fusion to promote transformation. In human PCa, there are about 25% patients have PTEN genomic deletion and T/E fusion events [50]. As shown here, PNT1a cells overexpressing WT p65 or active p536 alone cannot survive in mice although they could form colonies in soft agar. However, tumors are seen only when activated AKT is introduced into PNT1a-WT p65 and PNT1a-p65 S536E cells, with much larger tumor seen in the latter. Our data shows that cell group with active S536E expression exhibits significantly larger tumors and much higher tumor incidence than cell group with WT p65. Since both groups show similar level of total AKT and p65, as well as phosphorylated AKT 473 and 308 sites, the larger tumors and higher tumor incidence discovered must be driven by the only difference between these two groups the phosphorylation status of p65 ser536 site. Thus p65 Ser536, which is downstream of the T/E fusion gene, is sufficient to transform PNT1a cells with activated AKT signaling, strongly implicating this pathway is transformed by the T/E fusion gene in the context of T/E fusion gene expression. One should note, these PNT1a cells are simian virus 40 (SV40) immortalized normal prostate cells. In these cells p53 and Rb family of tumor suppressors are inactivated by the expression of the T antigen [53, 54]. Therefore, these down regulated tumor suppressors may also play a synergetic role in such fully transformed activities seen in mice. Future studies using other mouse model such as orthotopic or tail vein injection (or other cell lines) to examine the possible increased invasion *in vivo* is needed, which will further confirm p536's effects on motility as discovered by *in vitro* data.

A number of other pathways have also been implicated in transformation driven by the T/E fusion gene. T/E fusion gene expression is clearly altered by androgen receptor (AR) signaling, but PNT1a cells do not express AR so that alterations in AR signaling are clearly not required to transform immortalized prostate epithelial cells. As described above, our data strongly implicates Ser536 phosphorylation in cooperation with AKT activation in T/E fusion driven transformation. However, how Ser536 phosphorylation interacts with pathways implicated in T/E driven transformation such as Wnt pathway alterations, Sox 9 [52, 55] etc will require further investigation.

These novel discoveries above improve our understanding and characterization of T/E fusion expressing PCa which may expedite the practice of targeted therapy according to the specific genetic profiles of tumors. Additional genetically engineered mouse models will be needed for preclinical studies exploring combination therapies targeting T/E fusion, NF-kB, CCL2 and/or AKT pathways. If successful, such novel therapy strategy will benefit more than half of PCa patients who carry T/E fusions.

## MATERIALS and METHODS

### Cell culture

PNT1a and LNCaP cells were maintained in the RPMI with 10% fetal bovine serum (FBS) [9]. Human embryonic kidney-293 cell line 293t cells and 293-FT (Invitrogen) were cultured in the DMEM with 10% FBS.

Stable cell lines. Lentiviruses were obtained by collecting supernatant of 293-FT cells. PNT1a and LNCaP cells were infected by these viruses carrying WT p65, S536D, S536E and S536A. Stable cells were maintained in medium containing 200ug/ml G418.

### Site-directed mutagenesis

Single nucleotide mutagenesis was carried out according to the manufacturer's protocol (Stratagene, QuickChange Site-Directed Mutagenesis Kit). Briefly, primers with the target mutations were used in PCR to generate p536 site mutation S536D, S536E and S536A. Dpn1 enzyme was added to PCR products for 1 h in 37°C to digest template plasmid DNA before the transformation. Clones were sequenced to verify the mutations.

### Western blotting

Anti-p65 and anti-p536 were obtained from Cell Signaling Technology, Inc (Danvers, MA, USA) and were used at 1:1000 dilution for Western blotting using procedures described previously [56]. Blot signals were visualized using enhanced chemiluminescence (Thermo Fisher Scientific, Inc) and exposed and developed with films.

### Microarray and data analysis

Total RNA samples were extracted with RNeasy Mini Kit (Qiagen) according to the manufacture's recommendations. RNA quality was assessed with Nanodrop (Invitrogen). The cDNA reverse transcription and fluorescent labeling reactions were carried out using Invitrogen SuperScript Plus Direct cDNA Labeling System with Alexa Fluor S'-Aminohexylacrylamido-dUTP. Briefly, 2ug of aRNA and Universal Human Reference RNA (Stratagene) were labeled in reverse transcription with Alexa647 and Alexa 555 aha-dUTPs respectively. After labeling, 2ug of each aRNA was mixed with 2 ug of reference RNA and purified with Qiagen MiniElute PCR Purification kit according to manufacturer's instructions. Eluted sample was mixed with 10× Blocking Solution and 2× HiRPM buffer (Agilent Technologies), incubated for 2 min at 95°C and hybridized on 8× 60K Whole Human Genome Oligo Microarray chip (Agilent Technologies) using SureHyb DNA Microarray Hybridization Chambers in DNA Microarray hybridization oven (Agilent Technologies) at 10 rpm, 65°C for 18 h. After hybridization, slides were washed in Gene Expression Wash Buffer 1 and 2 for 1 min. Microarrays were scanned with a dynamic autofocus microarray scanner (Agilent Microarray Scanner–G2565BA, Agilent Technologies) using Agilent-provided parameters (Red and Green photomultiplier tube (PMT) were each set at 100%, and scan resolution was set to 3 μm). The Feature Extraction Software v9.1.3.1 (Agilent Technologies) was used to extract and analyze the signals. Expression arrays were processed and loess-normalized using BioConductor. Array data have been deposited in the public Gene Expression Omnibus (GEO) database (accession number GSE63210). For each experimental group, top differentially expressed genes relative to control were defined (using fold change>1.4 for each profile in a given p65 group—WT, S536A, or S536E—compared to each control profile), and the set of top differential genes found for any p65 group were clustered, using a supervised approach as described elsewhere [57]. Java TreeView (21) represented expression patterns as color maps.

### Quantitative real-time PCR

WT p65, CCL2 and β-actin primers were as described previously [2, 56]. 5 μl of the template cDNA (1:20 dilution) were used in a final reaction volume of 15ul. The Master mix for real time PCR contained 2mM MgCl2, 0.4 μM each forward and reverse primers and 7.5 μl of DNA Master SYBR GREEN (2X; ABI Company). Real-time PCR was done by using the ABI instrument from followed by a 3-step PCR protocol with different annealing temperatures and primers as shown in Supplementary Table 1. The relative expression was calculated by ΔCt among different experimental groups normalized to β-actin expression.

### Detection of secreted CCL2

1×10^6^ LNCaP cells expressing WT p65, S536D, S536E, S536A as well as vector control cells were plated in 60cm dishes in triplicate. Culture medium was collected 96 hour after seeding. 100ul of supernatant was used for detection of CCL2 protein level using Human CCL2 (MCP-1) ELISA Ready-SET-Go kit from ebioscience according to manufacturer's protocol. Experiments were repeated three times.

### Transfection and NF-kB reporter assays

Transient transfection was conducted in triplicate in 24-well plates using 293 cells as described previously [58]. The NF-kB luciferase reporter vector was obtained from Stratagene (Cat# 219077, PathDetect, Stratagene, La Jolla, CA). Luciferase activity was determined on triplicate samples and each experiment was repeated at least three times [56, 59].

### Proliferation assay

1×10^5^ cells were seeded on 24-well plates in triplicate and attached cells were counted using a cell counter as described previously [60]. The experiment was repeated three times. Final experiments were also confirmed by MTT assay using CellTiter 96 Non-Radioactive Cell Proliferation Assay from Promega (Cat #G4100; Promega, WI, USA).

### Soft agar assays

Five thousand PNT1a cells expressing either WT p65, S536D, S536E or S536A were mixed with the 0.7% agarose (top agar) and warm 2×RPMI 1640 + 20% fetal bovine serum and plated in each well of a 6-well plate on top of the prepared 1% base agar. Plates were incubated at 37°C for 14 d before the foci were stained with 0.005% Crystal Violet and counted.

### Matrigel invasion assay

The Matrigel invasion assays were conducted in triplicate as described previously [59]. Each experiment was repeated 3 times.

### CCL2 promoter construct

CCL2 promoter region (2812bp) was cloned into 12AB4KRC_CCL2Prom_pMK-RQ plasmid by Life technology. This clone was digested using KpnI and SmaI and the CCL2 promoter region into pGL3-Basic vector. Plasmid was sequenced and confirmed the accuracy before any further studies.

### NF-kB reporter assays

Transient transfection was conducted in triplicate in 24-well plates as described previously [58]. The NF-kB luciferase reporter vector was obtained from Stratagene (Cat# 219077, PathDetect, Stratagene, La Jolla, CA). Luciferase activity was determined on triplicate samples and each experiment was repeated at least three times.

### Immunohistochemistry

Immunohistochemistry (IHC) of VCaP subcutaneous tumors (Sh3 and sh (−)) was performed using anti-phospho-p65-Ser536 as described previously [11, 59]. P536 antibody was purchased from Cell Signaling (# 3031). Nuclear staining was quantified using a Vectra™-Inform™ image analysis system (Caliper Life Sciences, Hopkinton, MA). After segmenting nuclei, we scored IHC nuclear intensity into 4 bins as 0-3+ (0: no expression; 1+: low expression; 2+: moderate expression and 3+: high expression). InForm^TM^ was then used to quantitate the number of nuclei with staining intensity in each image. The percentage in each bin was then calculated. For all studies values compared using t-test or Mann-Whitney.

### Animal studies

Eight-week old SCID mice were used. 1×10^6^ PNT1a cells expressing various constructs in 50ul volume were mixed with 50ul matrigel (BD Bioscience, San Jose, CA, USA) and were injected subcutaneously over each lateral flank of mice (total of two sites per mouse). In our first study, we injected PNT1a cells overexpressing WT, active or dominant negative p65 to see if p536 can transform PNT1a cell *in vivo*. However, no obvious tumor growth was found in any group. Therefore, we introduced activated AKT, another well established factor which show strong effects on promoting PCa cell growth, into these PNT1a cells using lentiviruses carrying myristoylated AKT. The lentiviral vector carrying myristoylated human AKT1 (FU-S*AKT-CRW) was constructed in Dr.Witte's lab, which contains mRFP fluorescent marker. Details were described in ref [61, 62]. Lentiviruses were produced by standard protocol as described previously [9]. Cells were infected with virus and sorted by mRFP fluorescence. There were three groups of mice (10 mice / group; two injection sites per mouse.) injected with PNT1a-WT p65-AKT; PNT1a-p65 S536E+AKT and PNT1a control cells respectively. Tumor growth was monitored weekly. At end of 8 weeks, mice were euthanized and primary tumors were excised, weighed, and a portion of the tumor was frozen in liquid nitrogen for molecular analysis and another portion fixed and paraffin-embedded. Differences in mean tumor weight are examined by t-test. All procedures were approved by the Baylor College of Medicine Institutional Animal Use and Care Committee (IACUC protocol number #AN-6222).

## SUPPLEMENTARY MATERIAL AND TABLE



## References

[1] Soller M J, Isaksson M, Elfving P, Soller W, Lundgren R, Panagopoulos I (2006). Confirmation of the high frequency of the TMPRSS2/ERG fusion gene in prostate cancer. Genes Chromosomes Cancer.

[2] Wang J, Cai Y, Ren C, Ittmann M (2006). Expression of variant TMPRSS2/ERG fusion messenger RNAs is associated with aggressive prostate cancer. Cancer Res.

[3] Yoshimoto M, Joshua A M, Cunha I W, Coudry R A, Fonseca F P, Ludkovski O, Zielenska M, Soares F A, Squire J A (2008). Absence of TMPRSS2:ERG fusions and PTEN losses in prostate cancer is associated with a favorable outcome. Mod Pathol.

[4] Cai C, Wang H, Xu Y, Chen S, Balk S P (2009). Reactivation of androgen receptor-regulated TMPRSS2:ERG gene expression in castration-resistant prostate cancer. Cancer Res.

[5] Tomlins S A, Rhodes D R, Perner S, Dhanasekaran S M, Mehra R, Sun X W, Varambally S, Cao X, Tchinda J, Kuefer R, Lee C, Montie J E, Shah R B, Pienta K J, Rubin M A, Chinnaiyan A M (2005). Recurrent fusion of TMPRSS2 and ETS transcription factor genes in prostate cancer. Science.

[6] Demichelis F, Fall K, Perner S, Andren O, Schmidt F, Setlur S R, Hoshida Y, Mosquera J M, Pawitan Y, Lee C, Adami H O, Mucci L A, Kantoff P W, Andersson S O, Chinnaiyan A M, Johansson J E, Rubin M A (2007). TMPRSS2:ERG gene fusion associated with lethal prostate cancer in a watchful waiting cohort. Oncogene.

[7] Clark J, Merson S, Jhavar S, Flohr P, Edwards S, Foster C S, Eeles R, Martin F L, Phillips D H, Crundwell M, Christmas T, Thompson A, Fisher C, Kovacs G, Cooper C S (2006). Diversity of TMPRSS2-ERG fusion transcripts in the human prostate. Oncogene.

[8] Cerveira N, Ribeiro F R, Peixoto A, Costa V, Henrique R, Jeronimo C, Teixeira M R (2006). TMPRSS2-ERG gene fusion causing ERG overexpression precedes chromosome copy number changes in prostate carcinomas and paired HGPIN lesions. Neoplasia.

[9] Wang J, Cai Y, Yu W, Ren C, Spencer D M, Ittmann M (2008). Pleiotropic biological activities of alternatively spliced TMPRSS2/ERG fusion gene transcripts. Cancer research.

[10] Shao L, Tekedereli I, Wang J, Yuca E, Tsang S, Sood A, Lopez-Berestein G, Ozpolat B, Ittmann M (2012). Highly specific targeting of the TMPRSS2/ERG fusion gene using liposomal nanovectors. Clin Cancer Res.

[11] Wang J, Cai Y, Shao L J, Siddiqui J, Palanisamy N, Li R, Ren C, Ayala G, Ittmann M M (2010). Activation of NF-kB by TMPRSS2/ERG fusion isoforms through Toll-like receptor-4. Cancer research.

[12] Inoue J, Gohda J, Akiyama T, Semba K (2007). NF-kappaB activation in development and progression of cancer. Cancer Sci.

[13] Jin R, Yi Y, Yull F E, Blackwell T S, Clark P E, Koyama T, Smith J A, Matusik R J (2014). NF-kappaB gene signature predicts prostate cancer progression. Cancer Res.

[14] Shukla S, MacLennan G T, Fu P, Patel J, Marengo S R, Resnick M I, Gupta S (2004). Nuclear factor-kappaB/p65 (Rel A) is constitutively activated in human prostate adenocarcinoma and correlates with disease progression. Neoplasia.

[15] Jain G, Cronauer M V, Schrader M, Moller P, Marienfeld R B (2012). NF-kappaB signaling in prostate cancer: a promising therapeutic target?. World J Urol.

[16] Nguyen D P, Li J, Yadav S S, Tewari A K (2014). Recent insights into NF-kappaB signalling pathways and the link between inflammation and prostate cancer. BJU Int.

[17] Domingo-Domenech J, Mellado B, Ferrer B, Truan D, Codony-Servat J, Sauleda S, Alcover J, Campo E, Gascon P, Rovira A, Ross J S, Fernandez P L, Albanell J (2005). Activation of nuclear factor-kappaB in human prostate carcinogenesis and association to biochemical relapse. Br J Cancer.

[18] Jain G, Cronauer M V, Schrader M, Moller P, Marienfeld R B NF-kappaB signaling in prostate cancer: a promising therapeutic target?. World journal of urology.

[19] Jin R J, Lho Y, Connelly L, Wang Y, Yu X, Saint Jean L, Case T C, Ellwood-Yen K, Sawyers C L, Bhowmick N A, Blackwell T S, Yull F E, Matusik R J (2008). The nuclear factor-kappaB pathway controls the progression of prostate cancer to androgen-independent growth. Cancer Res.

[20] Kim J S, Roberts J M, Bingman W E, Shao L, Wang J, Ittmann M M, Weigel N L (2014). The Prostate Cancer TMPRSS2:ERG Fusion Synergizes With the Vitamin D Receptor (VDR) to Induce CYP24A1 Expression-Limiting VDR Signaling. Endocrinology.

[21] Vermeulen L, De Wilde G, Notebaert S, Vanden Berghe W, Haegeman G (2002). Regulation of the transcriptional activity of the nuclear factor-kappaB p65 subunit. Biochem Pharmacol.

[22] Viatour P, Merville M P, Bours V, Chariot A (2005). Phosphorylation of NF-kappaB and IkappaB proteins: implications in cancer and inflammation. Trends Biochem Sci.

[23] Shao L, Zhou Z, Cai Y, Castro P, Dakhov O, Shi P, Bai Y, Ji H, Shen W, Wang J (2013). Celastrol suppresses tumor cell growth through targeting an AR-ERG-NF-kappaB pathway in TMPRSS2/ERG fusion gene expressing prostate cancer. PLoS One.

[24] Sasaki C Y, Barberi T J, Ghosh P, Longo D L (2005). Phosphorylation of RelA/p65 on serine 536 defines an I{kappa}B{alpha}-independent NF-{kappa}B pathway. J Biol Chem.

[25] Hu J, Nakano H, Sakurai H, Colburn N H (2004). Insufficient p65 phosphorylation at S536 specifically contributes to the lack of NF-kappaB activation and transformation in resistant JB6 cells. Carcinogenesis.

[26] Hu J, Haseebuddin M, Young M, Colburn N H (2005). Suppression of p65 phosphorylation coincides with inhibition of IkappaBalpha polyubiquitination and degradation. Mol Carcinog.

[27] Wang J, Cai Y, Shao L J, Siddiqui J, Palanisamy N, Li R, Ren C, Ayala G, Ittmann M Activation of NF-{kappa}B by TMPRSS2/ERG Fusion Isoforms through Toll-Like Receptor-4. Cancer research.

[28] Rao D S, Bradley S V, Kumar P D, Hyun T S, Saint-Dic D, Oravecz-Wilson K, Kleer C G, Ross T S (2003). Altered receptor trafficking in Huntingtin Interacting Protein 1-transformed cells. Cancer cell.

[29] Pahl H L (1999). Activators and target genes of Rel/NF-kappaB transcription factors. Oncogene.

[30] Li X, Zhao Y, Tian B, Jamaluddin M, Mitra A, Yang J, Rowicka M, Brasier A R, Kudlicki A (2014). Modulation of gene expression regulated by the transcription factor NF-kappaB/RelA. J Biol Chem.

[31] Loberg R D, Day L L, Harwood J, Ying C, St John L N, Giles R, Neeley C K, Pienta K J (2006). CCL2 is a potent regulator of prostate cancer cell migration and proliferation. Neoplasia.

[32] Lu Y, Cai Z, Galson D L, Xiao G, Liu Y, George D E, Melhem M F, Yao Z, Zhang J (2006). Monocyte chemotactic protein-1 (MCP-1) acts as a paracrine and autocrine factor for prostate cancer growth and invasion. Prostate.

[33] Roca H, Varsos Z, Pienta K J (2008). CCL2 protects prostate cancer PC3 cells from autophagic death via phosphatidylinositol 3-kinase/AKT-dependent survivin up-regulation. J Biol Chem.

[34] Lin T H, Liu H H, Tsai T H, Chen C C, Hsieh T F, Lee S S, Lee Y J, Chen W C, Tang C H (2013). CCL2 increases alphavbeta3 integrin expression and subsequently promotes prostate cancer migration. Biochim Biophys Acta.

[35] Roebuck K A, Carpenter L R, Lakshminarayanan V, Page S M, Moy J N, Thomas L L (1999). Stimulus-specific regulation of chemokine expression involves differential activation of the redox-responsive transcription factors AP-1 and NF-kappaB. J Leukoc Biol.

[36] Wang J, Cai Y, Shao L J, Siddiqui J, Palanisamy N, Li R, Ren C, Ayala G, Ittmann M (2011). Activation of NF-{kappa}B by TMPRSS2/ERG Fusion Isoforms through Toll-Like Receptor-4. Cancer Res.

[37] Attard G, Clark J, Ambroisine L, Fisher G, Kovacs G, Flohr P, Berney D, Foster C S, Fletcher A, Gerald W L, Moller H, Reuter V, De Bono J S, Scardino P, Cuzick J, Cooper C S (2008). Duplication of the fusion of TMPRSS2 to ERG sequences identifies fatal human prostate cancer. Oncogene.

[38] Zhang J, Patel L, Pienta K J (2010). CC chemokine ligand 2 (CCL2) promotes prostate cancer tumorigenesis and metastasis. Cytokine Growth Factor Rev.

[39] Loberg R D, Ying C, Craig M, Yan L, Snyder L A, Pienta K J (2007). CCL2 as an important mediator of prostate cancer growth *in vivo* through the regulation of macrophage infiltration. Neoplasia.

[40] Tsaur I, Rutz J, Makarevic J, Juengel E, Gust K M, Borgmann H, Schilling D, Nelson K, Haferkamp A, Bartsch G, Blaheta R A (2014). CCL2 promotes integrin-mediated adhesion of prostate cancer cells *in vitro*. World J Urol.

[41] Sun T, Mary L G, Oh W K, Freedman M L, Pomerantz M, Pienta K J, Kantoff P W (2011). Inherited variants in the chemokine CCL2 gene and prostate cancer aggressiveness in a Caucasian cohort. Clin Cancer Res.

[42] Lu Y, Chen Q, Corey E, Xie W, Fan J, Mizokami A, Zhang J (2009). Activation of MCP-1/CCR2 axis promotes prostate cancer growth in bone. Clin Exp Metastasis.

[43] Zhang J, Lu Y, Pienta K J (2010). Multiple roles of chemokine (C-C motif) ligand 2 in promoting prostate cancer growth. J Natl Cancer Inst.

[44] Li X, Loberg R, Liao J, Ying C, Snyder L A, Pienta K J, McCauley L K (2009). A destructive cascade mediated by CCL2 facilitates prostate cancer growth in bone. Cancer Res.

[45] Loberg R D, Ying C, Craig M, Day L L, Sargent E, Neeley C, Wojno K, Snyder L A, Yan L, Pienta K J (2007). Targeting CCL2 with systemic delivery of neutralizing antibodies induces prostate cancer tumor regression *in vivo*. Cancer Res.

[46] Perkins N D (2006). Post-translational modifications regulating the activity and function of the nuclear factor kappa B pathway. Oncogene.

[47] Lawrence T, Bebien M, Liu G Y, Nizet V, Karin M (2005). IKKalpha limits macrophage NF-kappaB activation and contributes to the resolution of inflammation. Nature.

[48] Buss H, Dorrie A, Schmitz M L, Hoffmann E, Resch K, Kracht M (2004). Constitutive and interleukin-1-inducible phosphorylation of p65 NF-{kappa}B at serine 536 is mediated by multiple protein kinases including I{kappa}B kinase (IKK)-{alpha}, IKK{beta}, IKK{epsilon}, TRAF family member-associated (TANK)-binding kinase 1 (TBK1), and an unknown kinase and couples p65 to TATA-binding protein-associated factor II31-mediated interleukin-8 transcription. J Biol Chem.

[49] Adli M, Baldwin A S (2006). IKK-i/IKKepsilon controls constitutive, cancer cell-associated NF-kappaB activity via regulation of Ser-536 p65/RelA phosphorylation. J Biol Chem.

[50] Squire J A (2009). TMPRSS2-ERG and PTEN loss in prostate cancer. Nat Genet.

[51] Qu X, Randhawa G, Friedman C, Kurland B F, Glaskova L, Coleman I, Mostaghel E, Higano C S, Porter C, Vessella R, Nelson P S, Fang M (2013). A three-marker FISH panel detects more genetic aberrations of AR PTEN and TMPRSS2/ERG in castration-resistant or metastatic prostate cancers than in primary prostate tumors. PLoS One.

[52] Li Y, Kong D, Wang Z, Ahmad A, Bao B, Padhye S, Sarkar F H (2011). Inactivation of AR/TMPRSS2-ERG/Wnt signaling networks attenuates the aggressive behavior of prostate cancer cells. Cancer Prev Res (Phila).

[53] Rizzi F, Naponelli V, Silva A, Modernelli A, Ramazzina I, Bonacini M, Tardito S, Gatti R, Uggeri J, Bettuzzi S (2014). Polyphenon E(R), a standardized green tea extract, induces endoplasmic reticulum stress, leading to death of immortalized PNT1a cells by anoikis and tumorigenic PC3 by necroptosis. Carcinogenesis.

[54] Degeorges A, Hoffschir F, Cussenot O, Gauville C, Le Duc A, Dutrillaux B, Calvo F (1995). Recurrent cytogenetic alterations of prostate carcinoma and amplification of c-myc or epidermal growth factor receptor in subclones of immortalized PNT1 human prostate epithelial cell line. Int J Cancer.

[55] Cai C, Wang H, He H H, Chen S, He L, Ma F, Mucci L, Wang Q, Fiore C, Sowalsky A G, Loda M, Liu X S, Brown M, Balk S P, Yuan X (2013). ERG induces androgen receptor-mediated regulation of SOX9 in prostate cancer. J Clin Invest.

[56] Shao L, Zhou Z, Cai Y, Castro P, Dakhov O, Shi P, Bai Y, Ji H, Shen W, Wang J Celastrol suppresses tumor cell growth through targeting an AR-ERG-NF-kappaB pathway in TMPRSS2/ERG fusion gene expressing prostate cancer. PloS one.

[57] Creighton C J, Casa A, Lazard Z, Huang S, Tsimelzon A, Hilsenbeck S G, Osborne C K, Lee A V (2008). Insulin-like growth factor-I activates gene transcription programs strongly associated with poor breast cancer prognosis. J Clin Oncol.

[58] Cai Y, Wang J, Li R, Ayala G, Ittmann M, Liu M (2009). GGAP2/PIKE-a directly activates both the Akt and nuclear factor-kappaB pathways and promotes prostate cancer progression. Cancer Res.

[59] Wang J, Stockton D W, Ittmann M (2004). The fibroblast growth factor receptor-4 Arg388 allele is associated with prostate cancer initiation and progression. Clin Cancer Res.

[60] Polnaszek N, Kwabi-Addo B, Wang J, Ittmann M (2004). FGF17 is an autocrine prostatic epithelial growth factor and is upregulated in benign prostatic hyperplasia. Prostate.

[61] Xin L, Lawson D A, Witte O N (2005). The Sca-1 cell surface marker enriches for a prostate-regenerating cell subpopulation that can initiate prostate tumorigenesis. Proc Natl Acad Sci U S A.

[62] Xin L, Teitell M A, Lawson D A, Kwon A, Mellinghoff I K, Witte O N (2006). Progression of prostate cancer by synergy of AKT with genotropic and nongenotropic actions of the androgen receptor. Proc Natl Acad Sci U S A.

