# Supplementary material for: Function of phosphorylation of NF-kB p65 ser536 in prostate cancer oncogenesis
Source: Oncotarget. 2015 Jan 31;6(8):6281–94. doi: 10.18632/oncotarget.3366 (PMC4467437; doi:10.18632/oncotarget.3366)
Supplement: Supplementary file 1 [file oncotarget-06-6281-s001.pdf]

Function of phosphorylation of NF-kB p65 ser536 in prostate cancer oncogenesis

Supplementary Material

STable 1: Oligonucleotide primer sequences and reaction condition for real-time PCR

Primer name	primer sequnce	Annealing Temperatures
WFDC2 RTF	5’-CGGCTTCACCCTAGTCTCAG-3’	60^0C
WFDC2 RTR	5’-GGGCAGGAACCCTCCTTATC-3’	
IFI27 RTF	5’-CTCTGCCCGGTGTTTTTGTT-3’	60^0C
IFI27 RTR	5’-CTTTGGCCACACTGGTCACT-3’	
LCN2 RTF	5’-GTGGTATGTGGTAGGCCTGG-3’	60^0C
LCN2 RTR	5’-CACCACTCGGACGAGGTAAC-3’	
Cyclin D1 RTF	5’- CCGTCCATGCGGAAGATC-3’	60^0C
Cyclin D1 RTR	5’-GAAGACCTCCTCCTCGCACT-3’	
MMP-9 RTF	5’- GAGGCGCTCATGTACCCTATGT-3’	60^0C
MMP-9 RTR	5’- CCGTGGCTCAGGTTCAGG-3’	
IL8 RTF	5'- CTT GGC AGC CTT CCT GAT TT -3'	60^0C
IL8RTR	5'- TTC TTT AGC ACT CCT TGG CAA AA -3'	
